# POLICY SERIES: THE HRSA GERIATRICS EDUCATION INITIATIVES: A POLICY AND PROGRAM UPDATE

**DOI:** 10.1093/geroni/igz038.1359

**Published:** 2019-11-08

**Authors:** Catherine Carrico, Kathryn Hyer, Brian W Lindberg

**Affiliations:** 1 University of Wyoming, Laramie, Wyoming, United States; 2 University of South Florida, Tampa, Florida, United States; 3 The Gerontological Society of America, Washington, District of Columbia, United States

## Abstract

This session will address the evolving work of Geriatric Workforce Enhancement Programs (GWEP) to educate and train the primary care workforce and partner with community based organizations to address gaps in healthcare. The inaugural GWEP awardees were announced at the 2015 White House Conference on Aging. In November 2018 the Health Resources and Services Administration (HRSA), released the second GWEP Notice of Funding Opportunity (NoFO). The new funding opportunity reflects current policy priorities. In addition, for the first time in 9 years a Notice of Funding Opportunity was released for the Geriatric Academic Career Award (GACA) program. The return of the GACA program indicates the success of advocacy efforts to increase programs to support geriatric training and the development of geriatric academic professionals. Individual symposiums will explore the policy priorities reflected in the GWEP NoFO including the use of technology for training and care delivery, the age-friendly healthcare and dementia-friendly community initiatives, and value-based care including improving partnership with community based organizations. Additionally, an individual symposium will present the GACA program and discuss the advocacy process leading to the reintroduction of this program. During the last congress advocates of the GWEP and GACA programs supported authorization and funding of geriatric programs. Geriatrics programs describing the work of the GWEPs and GACAs were reinserted into the legislative process, although this reauthorization was not completed. This advocacy process garnered strong bipartisan and agency support. The session will describe these efforts and will conclude with an update on current authorization and appropriation status.

